# *Entamoeba* Chitinase is Required for Mature Round Cyst Formation

**DOI:** 10.1128/spectrum.00511-21

**Published:** 2021-08-04

**Authors:** Fumika Mi-ichi, Miako Sakaguchi, Shinjiro Hamano, Hiroki Yoshida

**Affiliations:** a Division of Molecular and Cellular Immunoscience, Department of Biomolecular Sciences, Faculty of Medicine, Saga University, Saga, Japan; b Central Laboratory, Institute of Tropical Medicine (NEKKEN), Nagasaki University, Nagasaki, Japan; c The Joint Usage/Research Center on Tropical Disease, Institute of Tropical Medicine (NEKKEN), Nagasaki University, Nagasaki, Japan; d Department of Parasitology, Institute of Tropical Medicine (NEKKEN), Nagasaki University, Nagasaki, Japan; Institut Pasteur

**Keywords:** amoebiasis, infectious disease, chitin, dormancy, encystation

## Abstract

Entamoeba histolytica, a protozoan parasite, causes amoebiasis in humans. Amoebiasis transmission is solely mediated by chitin-walled cysts, which are produced in the large intestine of humans from proliferative trophozoites by a cell differentiation process called encystation. Resistance to environmental stresses, an essential characteristic for transmission, is attributed to the cyst wall, which is constructed from chitin and several protein components, including chitinase. Chitinase may play a key role in cyst wall formation; however, this has not been confirmed. Here, to elucidate the physiological role of chitinase during *Entamoeba* encystation, we identified a new chitinase inhibitor, 2,6-dichloro-4-[2-(1-piperazinyl)-4-pyridinyl]-*N*-(1,3,5-trimethyl-1*H*-pyrazol-4-yl)-benzenesulfonamide, by recombinant-*Entamoeba* chitinase-based screening of 400 Pathogen Box chemicals. This compound dose dependently inhibited native chitinase associated with Entamoeba invadens encystation, a model for E. histolytica encystation, with an 50% inhibitory concentration (IC_50_) of ∼0.6 μM, which is comparable to the IC_50_s (0.2 to 2.5 μM) for recombinant E. histolytica and E. invadens chitinases. Furthermore, the addition of this compound to E. invadens encystation-inducing cultures increased the generation of cyst walls with an abnormal shape, the most characteristic of which was a “pot-like structure.” A similar structure also appeared in standard culture, but at a far lower frequency. These results indicate that chitinase inhibition increases the number of abnormal encysting cells, thereby significantly reducing the efficiency of cyst formation. Transmission electron microscopy showed that compound-treated encysting cells formed an abnormally loose cyst wall and an unusual gap between the cyst wall and cell membrane. Hence, *Entamoeba* chitinase is required for the formation of mature round cysts.

**IMPORTANCE** Amoebiasis is caused by Entamoeba histolytica infection and is transmitted by dormant *Entamoeba* cells or cysts. Cysts need to be tolerant to severe environmental stresses faced outside and inside a human host. To confer this resistance, *Entamoeba* parasites synthesize a wall structure around the cell during cyst formation. This cyst wall consists of chitin and several protein components, including chitinase. The physiological roles of these components are not fully understood. Here, to elucidate the role of chitinase during cyst formation, we identified a new chitinase inhibitor by screening a library of 400 compounds. Using this inhibitor, we showed that chitinase inhibition causes the formation of abnormal cyst walls, the most characteristic of which is a “pot-like structure.” This results in decreased production of mature cysts. Chitinase is therefore required for *Entamoeba* to produce mature cysts for transmission to a new host.

Entamoeba histolytica, a protozoan parasite belonging to the phylum Amoebozoa, is the causative agent of amoebiasis, which is a global public health problem. The life cycle of E. histolytica alternates between two forms. One is the trophozoite, which proliferates and invades host cells and tissues, causing pathogenesis and typical symptoms of dysentery and amoebic liver abscesses (1, 2). The other is the cyst, which enables dormancy. This characteristic is essential for the parasite to be transmitted to a new host. Cysts differentiate from proliferative trophozoites via the transition process, encystation (1, 3, 4). E. histolytica is not able to encyst in *in vitro* culture; therefore, Entamoeba invadens, a reptilian parasite, has been generally adopted as a model for E. histolytica encystation (4, 5). *In vitro* cultures of E. invadens produce cysts that are able to hatch into trophozoites by the process of excystation (3, 4, 6). Therefore, this model system enables changes in cell morphology, components, and metabolism to be monitored during encystation and excystation, the two critical transition states in the *Entamoeba* life cycle.

During *Entamoeba* encystation, remarkable changes occur; motile amoeboid trophozoites become nonmotile, round, chitin-walled cysts. Mature cysts are resistant to environmental assaults outside the host, such as desiccation, as well as inside the host, such as strong acidic conditions (4, 7–9). Cyst morphology also prevents the loss of essential biomolecules. Major components of the cyst wall are chitin fibrils, Jacob and Jessie lectins, and chitinase (10, 11). The latter three components all have a chitin binding domain (CBD) that has been biochemically shown to bind chitin beads (11–15). All cyst wall components are stage specifically synthesized and layered around the encysting cells, forming a well-organized architecture (11, 13); a “wattle and daub” model has been proposed (7, 13).

Chitinase (EC 3.2.1.14) hydrolyzes chitin, a β-1,4-linked polymer of *N*-acetyl d-glucosamine. *Entamoeba* chitinase is suggested to be involved in remodeling the cyst wall during encystation (11, 12), but evidence for this is scarce. Among the most appropriate approaches to investigate *Entamoeba* chitinase physiology is pharmacological blockade. Allosamidin was previously shown to inhibit the chitinase activities of partially purified fractions from E. invadens cyst lysate, but it did not impair cyst formation, although it delayed progression (16, 17). However, this available inhibitor has become difficult to obtain commercially. Furthermore, studies for other chemicals that inhibit *Entamoeba* chitinase were not reported.

Here, to overcome this limitation, we performed a recombinant *Entamoeba* chitinase-based screen of the 400 chemicals in the Pathogen Box from the Medicines for Malaria Venture (MMV; https://www.mmv.org/mmv-open/archived-projects/pathogen-box). We identified one compound that inhibited chitinase and analyzed its effects on the cyst formation process of E. invadens to elucidate *Entamoeba* chitinase physiology.

## RESULTS

### *In vitro* chitinase activity assay-based screening of the 400 Pathogen Box compounds.

**FIG 1:**
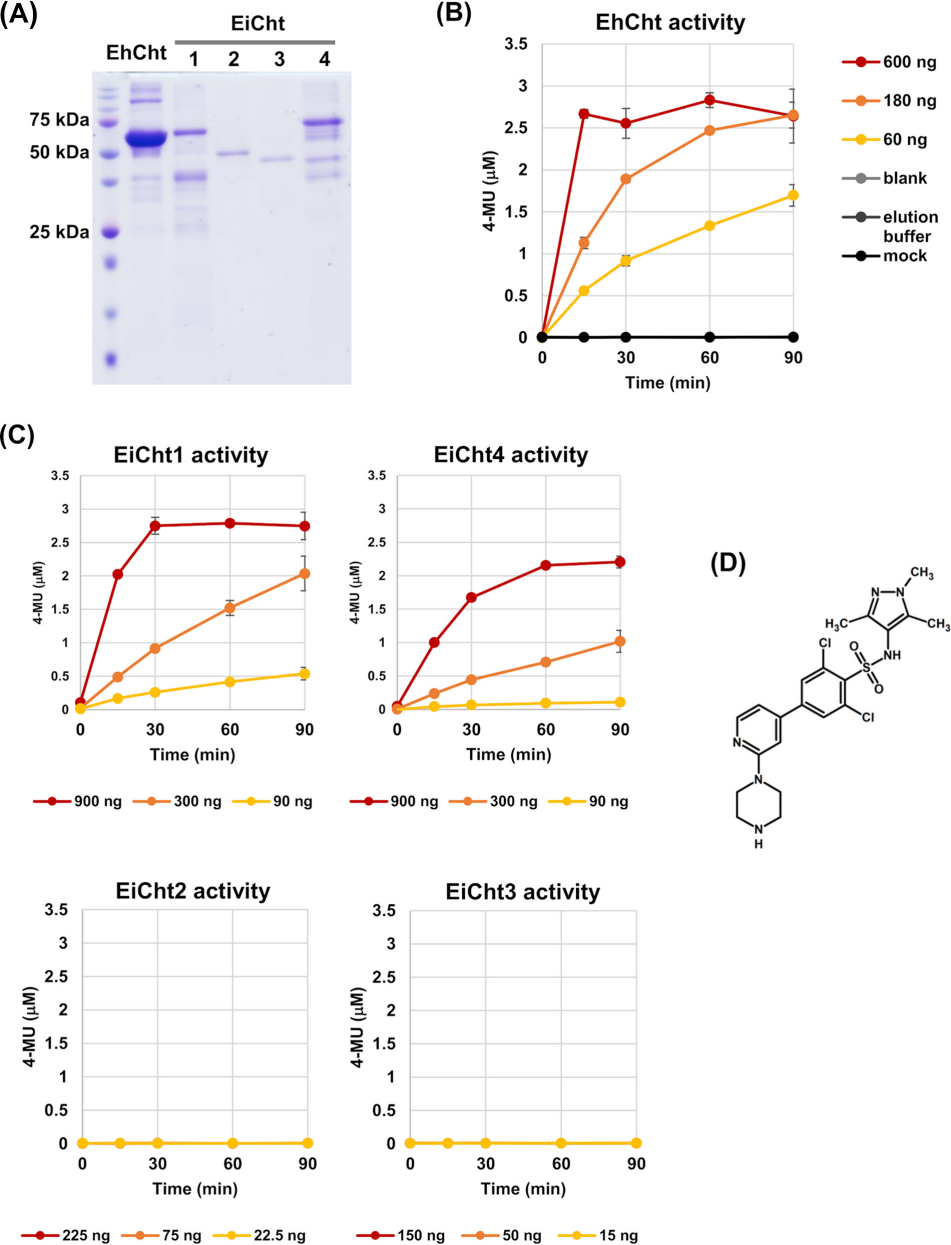
Establishment of an *in vitro* 96-well-plate-based chitinase activity assay. (A) SDS-PAGE of affinity-purified recombinant proteins. (B, C) Dose-dependent effects of rEhCht (B) and rEiCht1 and rEiCht4 (C) on chitinase activity. The data from three controls were consistent in the experiments whose results are shown in panels B and C and are only shown in panel B for clarity. Representative data of recombinant chitinase activities from three independent preparations are shown. Error bars show standard deviations. (D) Chemical structure of D-B-09.

In AmoebaDB (http://amoebadb.org/amoeba/), one gene, *EhCht* (EHI_109890), is annotated as a chitinase in the E. histolytica genome, whereas four genes, *EiCht1* to -*4* (EIN_404540, EIN_085570, EIN_096870, and EIN_136020, respectively) are annotated as chitinases in the E. invadens genome (11, 18, 19). All five chitinases possess conserved catalytic domains, which are phylogenetically related; however, two E. invadens chitinases (EiCht2 and -3) lack a chitin binding domain (CBD) (Fig. S1 in the supplemental material) (11, 18). We expressed all five chitinases as recombinant histidine (His)-tagged full-length proteins without signal peptide. The purified recombinant enzymes with a CBD (rEhCht, rEiCht1, and rEiCht4) dose dependently increased the production of 4-methylumbelliferone (4-MU) by hydrolyzing 4-methylumbelliferyl β-d-*N*,*N*′,*N*′′-triacetylchitotrioside [4MU-(GlcNAc)^3^], while the CBD-less recombinant enzymes (rEiCht2 and -3) did not (Fig. 1A to C), indicating the importance of the CBD for chitin hydrolysis. We determined the specific activities of purified recombinant *Entamoeba* chitinases to be as follows: EhCht, 58.0 ± 8.2 μmol/min/mg protein (mean ± standard deviation [SD]); EiCht1, 11.7 ± 5.3 μmol/min/mg protein; and EiCht4, 6.3 ± 1.1 μmol/min/mg protein. All three controls, including a mock (empty vector control), a solvent (elution buffer used in affinity chromatography), and a blank (50 mM Tris/HCl buffer [pH 8.0]) solution, gave almost constant levels of 4-MU within the time period monitored (Fig. 1B).

To screen the 400 Pathogen Box compounds, we employed a chitinase activity assay using the fluorogenic substrate 4MU-(GlcNAc)^3^ in a 96-well-plate format and set two assay conditions. One was for rEhCht, in which the reaction proceeded for 30 min at 37°C, and the other was for rEiCht1 and -4, in which the reaction proceeded for 60 min at 26°C. The amount of each enzyme in a well was in the range that produces 3 to 6 nmol 4-MU per min; for instance, 0.075 μg rEhCht, 0.45 μg rEiCht1, and 0.90 μg rEiCht4. We then screened the 400 compounds of the Pathogen Box; each compound was assayed at a final concentration of 10 μM. Dimethyl sulfoxide (DMSO) was used as a solvent control at 1% (vol/vol), the final DMSO concentration in all wells.

Three compounds, B-H-02, D-A-09, and D-B-09, inhibited the chitinase activity (≥75%) of at least one recombinant enzyme among rEhCht, rEiCht1, and rEiCht4 (Table 1; Fig. S2). The effects of each of these three compounds at 10 μM on E. histolytica trophozoite proliferation and E. invadens cyst formation were determined separately using a flow cytometry method described in reference 20 (Table 1). Among the three compounds, D-B-09 did not affect trophozoite proliferation but was a potent inhibitor of cyst formation. Therefore, D-B-09 was selected for further analysis (the structure of D-B-09 is shown in Fig. 1D). Thereafter, the compound was obtained from a commercial source (Cayman Chemical, Ann Arbor, MI, USA) to ensure its supply. The 50% inhibitory concentrations (IC_50_s) of D-B-09 for rEhCht, rEiCht1, and rEiCht4 were determined to be 0.3 ± 0.1, 0.8 ± 0.2, and 2.5 ± 1.3 μM (*n* = 3), respectively.

**TABLE 1:** Potencies of three screened compounds for inhibition of recombinant chitinases, trophozoite proliferation, and cyst wall formation

Plate, compound (10 μM)	Avg Cht activity ± SD (%)^*a*^			Avg value ± SD (%) for^*b*^:	
	EhCht	EiCht1	EiCht4	Cyst formation rate	Live trophozoites
D, B09	7.9 ± 4.4	25.0 ± 0.4	24.1 ± 2.6	55.9 ± 10.1	107 ± 2.5
B, H02	56.5 ± 7.5	26.8 ± 5.4	24.9 ± 1.2	25.6 ± 2.7	43.0 ± 8.8
D, A09	21.0 ± 6.1	65.3 ± 1.6	50.5 ± 3.3	96.1 ± 5.4	96.2 ± 13.6

a rCht activities relative to the value for the DMSO control were determined by three independent experiments.

b Cyst formation rates and live-trophozoite levels are from reference 20.

### Characterization of native chitinase associated with *E. invadens* encystation.

**FIG 2:**
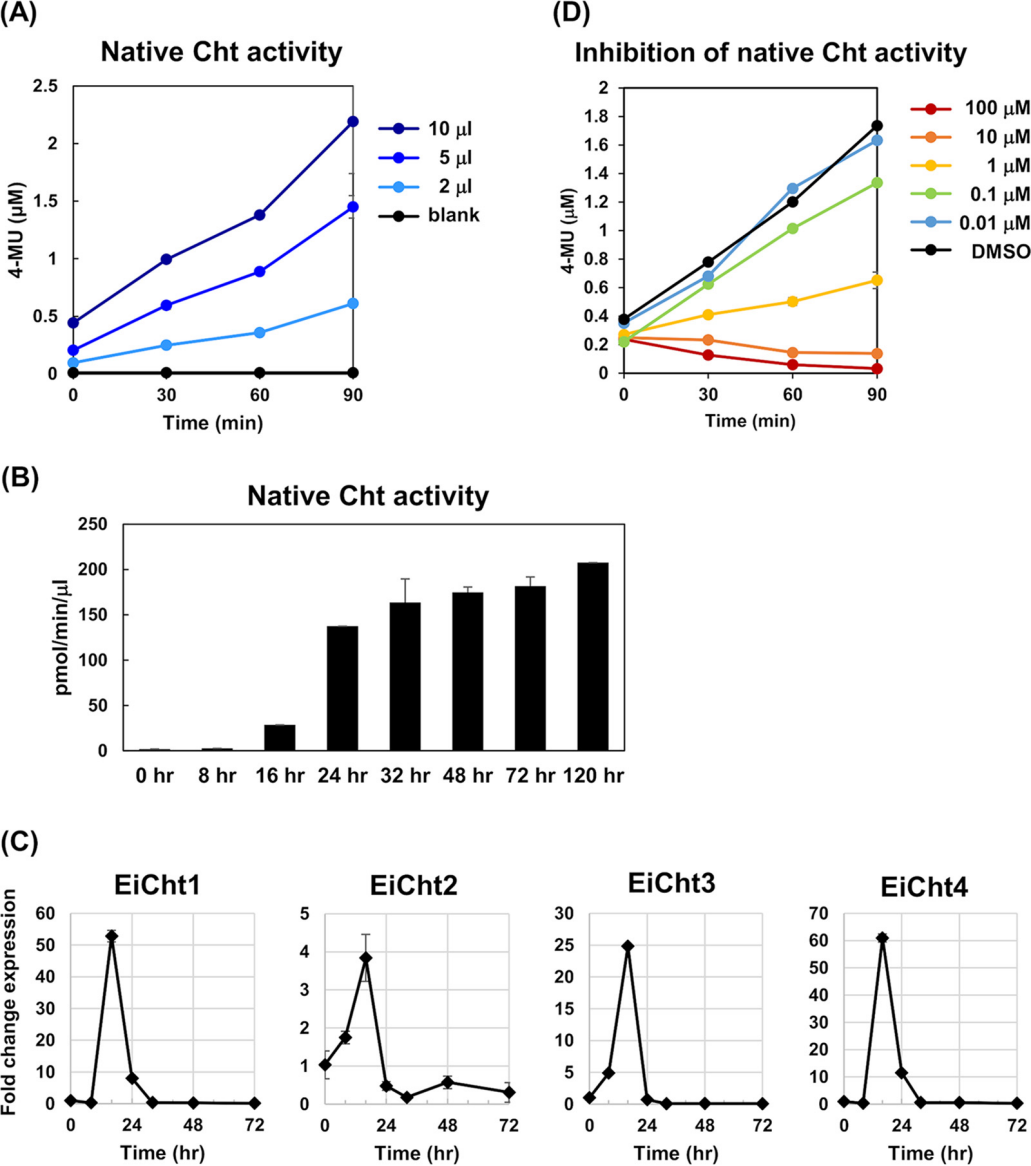
Characterization of native chitinase associated with encysting E. invadens cells. (A) Dose-dependent effects of native chitinase associated with encysting cells at 72 h postinduction. Encysting-cell suspensions were used as enzyme sources, and the final cell density in suspension could be deduced to be 1 × 10^7^ cells/ml (see Materials and Methods). Encysting-cell suspensions were also used in the experiments whose results are shown in panels B and D. (B) Profile of E. invadens cell-associated chitinase activity during encystation. (C) Transcriptional changes of the genes encoding EiCht1 to -4 during encystation. Expression levels are shown as fold changes at the indicated time points after the induction of encystation relative to the level at time zero h. Experiments were performed in triplicate. (D) Dose-dependent inhibition of encysting E. invadens cell-associated chitinase activity by D-B-09. Five microliters of encysting E. invadens cell suspension, which was prepared at 72 h postinduction, was used as the enzyme source. Representative data from three independent experiments are shown. Error bars show standard deviations.

Chitinase, a secreted enzyme, is an E. invadens cyst wall component (11, 21). Its activity was measured using encysting cells collected from the inducing culture. Encysting cells collected at 72 h postinduction hydrolyzed 4MU-(GlcNAc)^3^ and released 4-MU in a dose-dependent manner at 26°C (Fig. 2A). In contrast, trophozoites prepared from E. invadens cultures immediately after encystation induction showed almost no 4MU-(GlcNAc)^3^ hydrolyzing activity (time zero h in Fig. 2B). The cell-associated hydrolysis activity increased with time during encystation; in particular, a dramatic increase was observed between 16 and 24 h (Fig. 2B). Consistently, significant upregulation of *EiCht1* and *EiCht4* transcripts was observed at 16 h after induction (Fig. 2C). Notably, the elevation of encysting E. invadens cell-associated chitinase activity was well correlated with the time frame in which the formation of chitin fibrils in the cyst wall was visualized by a combination of flow cytometry and electron microscopy analysis (22). Furthermore, the hydrolysis of 4MU-(GlcNAc)^3^ associated with encysting E. invadens cells was sensitive to D-B-09 with an IC_50_ of 0.6 ± 0.2 μM (*n* = 3) (Fig. 2D), which was comparable to the IC_50_s for rEiCht1 and -4 (0.8 ± 0.2 and 2.5 ± 1.3 μM, respectively).

### Chitinase is required for the formation of round mature cysts during *Entamoeba* encystation.

**FIG 3:**
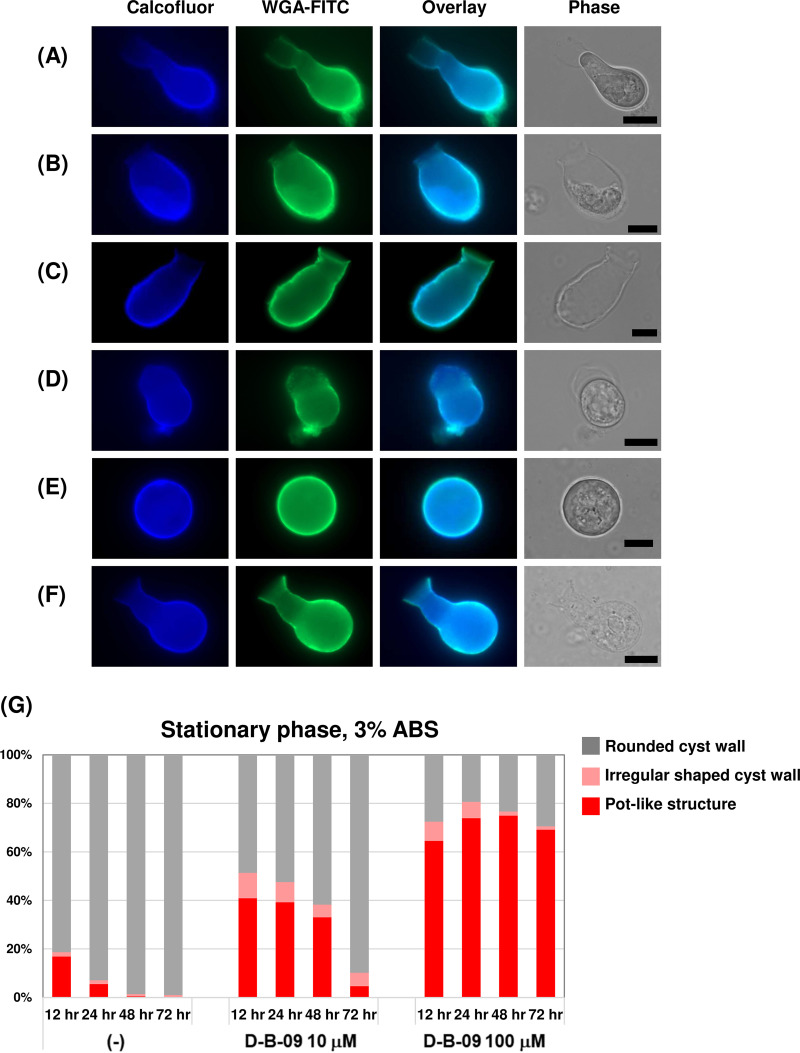
Effects of D-B-09 on E. invadens cyst formation. (A to F) Cell morphologies observed by fluorescence microscopy at 16 h after induction of encystation. Cells were sampled from the 10 μM D-B-09-treated encystation-inducing culture (A to E) or from the control culture (F). Cyst wall with pot-like structure associated with a cell (A, B, F) and not associated with a cell (C); irregularly shaped cell (D); and normal encysting cell (E). Scale bars indicate 10 μm. (G) Stacked bar graph of percentages of round, irregularly shaped, and pot-like cyst walls, which were detected at 12, 24, 48, and 72 h after induction of encystation. At each time point, 200 calcofluor-fluorescent cells were randomly selected and the number of cells with each structure counted. Representative data from three independent experiments are shown.

We then investigated the physiological role of chitinase during *Entamoeba* encystation. As shown by the results in Fig. 2D, the encysting E. invadens cell-associated chitinase activity was almost completely inhibited by 10 μM D-B-09. We therefore analyzed the effect of D-B-09 (10 and 100 μM) on the morphology of encysting E. invadens cells by fluorescence microscopy using fluorescein isothiocyanate (FITC)-conjugated wheat germ agglutinin (WGA) and calcofluor, both of which stain chitin, a major component of the cyst wall (13, 20). From 12 h post-encystation induction, D-B-09 treatment caused a high frequency of abnormal cyst wall structures that resembled a pot (hereinafter called “pot-like structure”). The pot-like structure existed in three forms; one was associated with live cells (Fig. 3A), another was associated with dead cells (Fig. 3B), and the third was free from cells (Fig. 3C). Cells with an irregularly shaped cyst wall (Fig. 3D) were also observed. In contrast, in the control culture, most cells were surrounded by a round cyst wall, which is the typical appearance of mature cysts (Fig. 3E). However, pot-like structures were also observed in the control culture (Fig. 3F), but at low frequency. Of note, although both dyes distinguishably stained all types of cyst wall structures, WGA staining visualized the wall structures in more detail than calcofluor staining, particularly the edges of the pot-like structures (Fig. 3A to D and F). The difference in visualization of the cyst wall structures became more substantial when a three-dimensional (3-D) model of a pot-like structure was made by confocal microscopy (Movie S1).

We then determined the time course of changes for each of the three cyst wall structures, i.e., pot-like, irregularly shaped, and round, by manual counting under a fluorescence microscope. The results are shown as stacked bar graphs (Fig. 3G). In the 10 μM D-B-09-treated culture, the percentage of pot-like structures was always higher than in the untreated control culture at the time points analyzed during encystation. In the treated culture, the percentage of pot-like structures was highest at 12 h and then decreased with time. This decrease seemed to result from disappearance of the pot-like structures, probably due to degradation, because total cell numbers in the treated culture decreased significantly after 48 h. The percentage of pot-like structures was elevated at all time points analyzed when the concentration was increased to 100 μM. Meanwhile, in the untreated control culture, the percentage of pot-like structures determined at 12 h was less than half that of the treated culture and became smaller with time. From 48 h onward, the effect of D-B-09 became more evident; in the control culture, mature cysts were dominantly observed, whereas in the 10 μM D-B-09-treated culture, significant numbers of pot-like structures could still be found (Fig. 3G). Despite the trends described above, the effect of D-B-09 on the production of pot-like structures fluctuated depending on the cell conditions used and the serum concentration in the encystation-inducing culture (Fig. 3G; Fig. S3). The effect of D-B-09 on cyst formation was influenced similarly (Table S1). The percentages of pot-like structures and the IC_50_s for cyst formation appeared to be related; the greater the number of pot-like structures generated, the lower the IC_50_ for cyst formation (Fig. 3G; Fig. S3 and Table S1).

**FIG 4:**
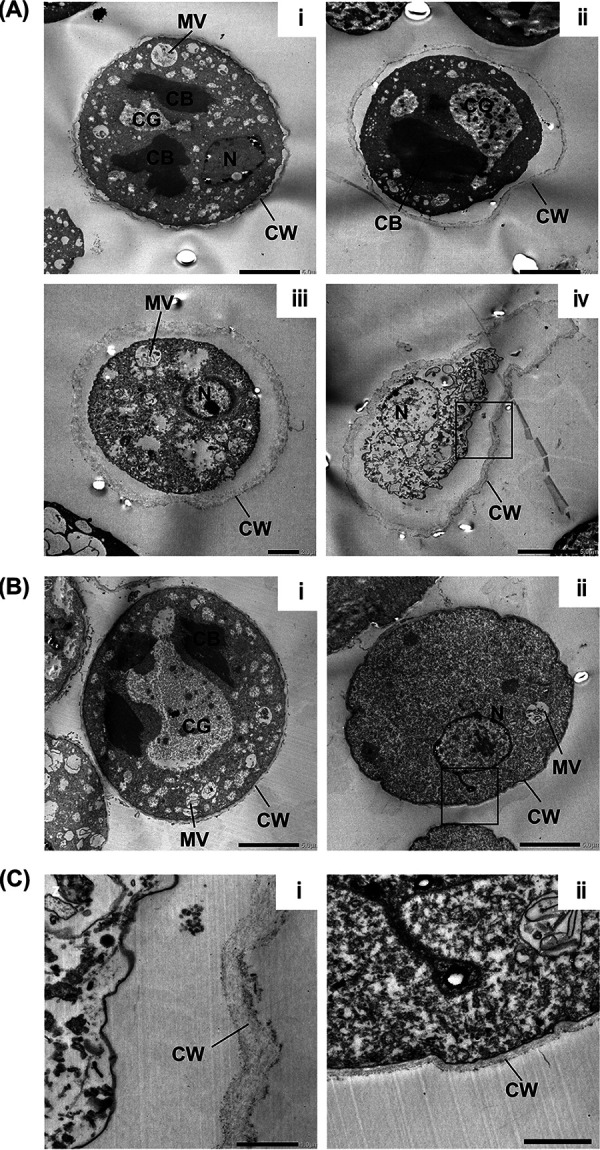
Transmission electron microscopy images. D-B-09-treated (10 μM) (A) or untreated control (B) encysting E. invadens cells observed at 24 h after induction of encystation. Four and two representative images from more than 30 and 20 cells in two independent experiments are shown in panels A and B, respectively. (C) Enlarged images of the boxed regions in panels Aiv and Bii are shown in panels i and ii, respectively. CB, chromatoid body; CW, cyst wall; MV, multivesicular body; N, nucleus. Scale bars indicate 5 μm in panels Ai, Aii, and Aiv, 2 μm in panels Bi and Bii, and 1 μm in panels Aiii, Ci, and Cii, respectively.

**FIG 5:**
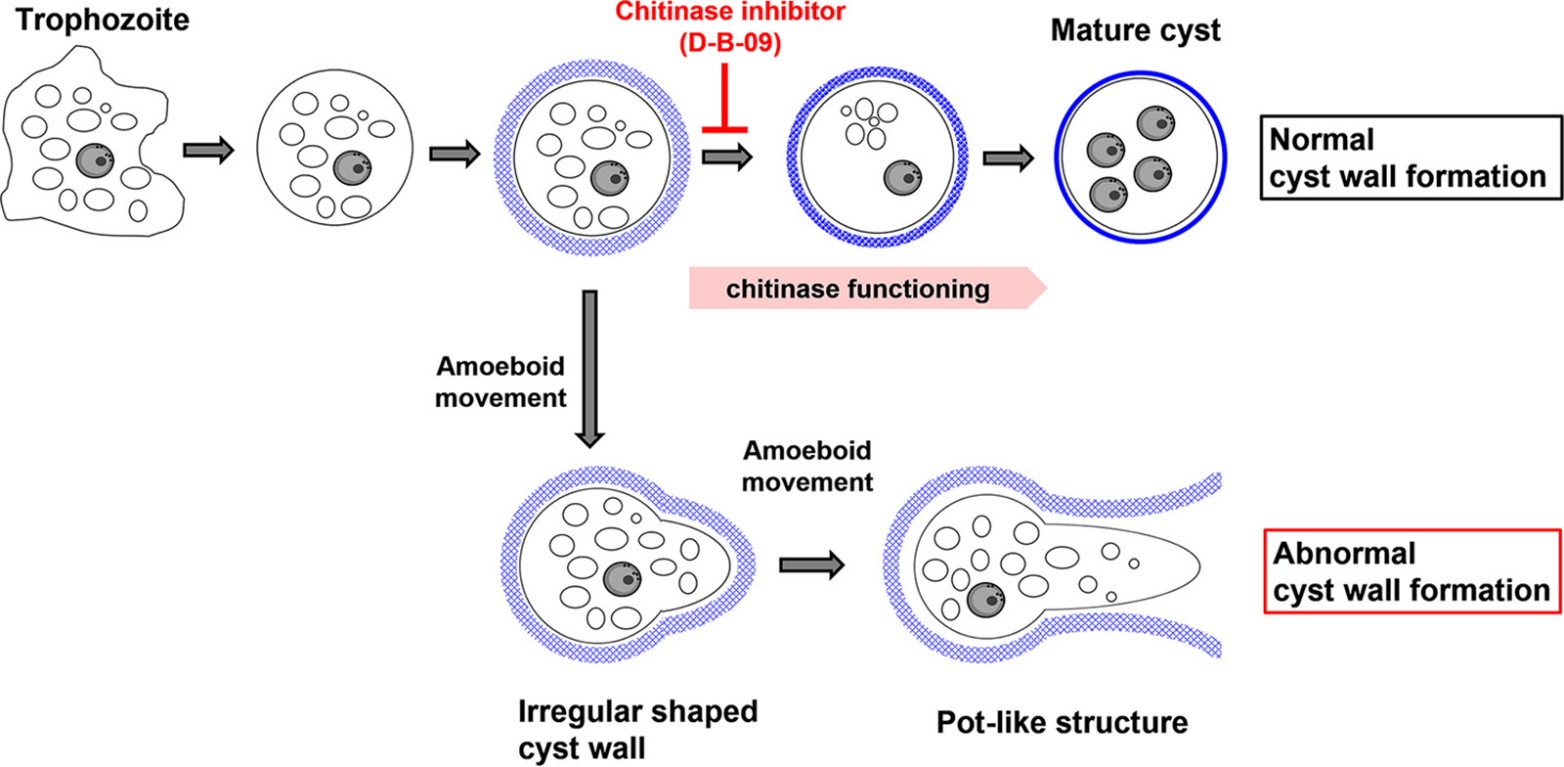
Proposed pathways of *Entamoeba* cyst wall formation.

To further determine the structural changes induced by D-B-09 treatment, we performed transmission electron microscopy analysis of encysting cells either in the presence or absence of D-B-09 at 24 h after induction (Fig. 4). In D-B-09-treated cells, the cell membrane was dissociated from the cyst wall (Fig. 4Ai to iv). The morphology of the cyst wall observed in the treated culture was fragmented and not compressed (Fig. 4Ai to iv and Ci) compared with that in untreated control cultures (Fig. 4Bi and ii and Cii). Pot-like structures encompassing unstructured cells, possibly dead cells, were also observed (Fig. 4Aiv). Importantly, in D-B-09-treated cells, the formation of chromatoid bodies, multivesicular bodies, and large-sized cytoplasmic glycogen, all structures that appear during normal cyst formation (22), was seen in encysting cells surrounded by the abnormal cyst wall (Fig. 4Ai and ii). These results indicate that changes to intracellular organelles proceed normally in encysting cells even when the cells do not form appropriate cyst walls (Fig. 5).

Taken together, these results indicate that pot-like structures and irregularly shaped cyst walls, both of which possess untightened chitin fibrils, are formed naturally during *Entamoeba* encystation and that their formation is enhanced by inhibition of chitinase activity.

## DISCUSSION

The formation of round, cyst-walled cells is a crucial process for *Entamoeba* to maintain its parasitic life cycle because mature cysts are the sole form able to be transmitted to a new host (4). The cyst wall is an essential structure that protects against environmental stresses. It is composed of chitin fibrils and chitin binding proteins, including chitinase (11). Chitinase is likely to play an important role in remodeling chitin during *Entamoeba* cyst wall formation (7, 11–13); however, this requires confirmation. Here, to elucidate *Entamoeba* chitinase physiology, we identified a new inhibitor, D-B-09, by recombinant-*Entamoeba* chitinase-based screening of 400 Pathogen Box compounds. The recombinant chitinases used for this screening system were synthesized in Escherichia coli as full-length proteins without signal peptide;s therefore, they may be a mixture of proteins correctly or incorrectly folded, including disulfide bonds in their CBD (14). Meanwhile, rEiCht2 and -3 did not show chitinase activity, which is inconsistent with a previous study showing their activities (23), and the reason needs to be addressed. Importantly, the chemical identified by the recombinant-enzyme-based screening system inhibited chitinase activity associated with encysting E. invadens cells dose dependently, with an IC_50_ comparable to those of recombinant *Entamoeba* chitinases.

The addition of the above-described inhibitor to encystation-inducing cultures significantly elevated the numbers of encysting cells that carried abnormal pot-like structures and irregularly shaped cyst walls. Such abnormal cyst walls, in particular pot-like structures, were also observed, but at much lower frequencies, in non-inhibitor-treated encystation-inducing cultures. These observations led us to consider that abnormal cyst walls are naturally formed during the cyst formation process and that abnormal chitinase activity may be a reason for this.

Electron microscopy showed that inhibition of *Entamoeba* chitinase by D-B-09 not only impaired the formation of the electron-dense cyst wall but also generated an unusual space between the cell membrane and the swollen cyst wall. These results indicate that chitinase activity is required for formation of the compressed cyst wall, which contains bundled chitin fibers and tight connections between the cell membrane and the cyst wall. Therefore, in the pot-like structures observed by fluorescence microscopy, we presume that the cyst wall of D-B-09-treated encysting *Entamoeba* cells might be too loose and weak to maintain a round cell morphology and that the pot-like structure results from amoeboid movement of the encysting cell inside the unusual cyst wall (Fig. 5). Chitinase may remodel chitin fibers, as previously predicted (11, 12). In addition, other factors that can bind to the chitin fibers, such as Jacob and Jessie proteins, are likely to play important roles in forming round, sealed cyst walls.

D-B-09 impaired E. invadens cyst formation with an IC_50_ of 1.4 ± 0.3 μM but showed almost no effect on E. histolytica or E. invadens trophozoite proliferation, with IC_50_s of >100 μM. The IC_50_ for cyst formation was obtained using culture conditions in which “stationary-phase” trophozoites (see Materials and Methods) were induced for encystation and cultivated in 3% adult bovine serum (ABS)-supplemented medium. Changing these conditions gave different IC_50_s (Table S1). Culture conditions can influence the consequences of a biological assay; in this case, the IC_50_ of D-B-09 for cyst formation and the percentage of pot-like structures generated were affected (Fig. 3G; Fig. S3 and Table S1). However, it is worth mentioning that the sensitivity of E. invadens cell-associated chitinase activity to D-B-09 did not fluctuate regardless of the cell conditions used and the serum concentration in the encystation-inducing culture (Table S2). Although a delay was seen in the stationary-phase-trophozoite samples, the overall time course of increased native chitinase activity was not affected (Fig. S4). Considering these results, the varied effects of D-B-09 treatment may reflect microlocalization of *Entamoeba* chitinase in the cyst wall and/or different structural features, such as chitin fibers and cyst wall proteins, which are potentially induced by different cell and culture conditions. Therefore, determination of the precise localization and movement of *Entamoeba* chitinase in the cyst wall and of detailed structural features of the lengths and the terminal sugars of chitin molecules and their modes of binding with other cyst wall components would substantiate the role of chitinase. In addition, the molecular mechanisms by which encystation is delayed by trophozoite conditions warrant further investigation.

Off-target effects of D-B-09 on *Entamoeba* cyst formation must be considered, because D-B-09 was originally isolated as an inhibitor of Trypanosoma brucei *N*-myristoyltransferase (NMT) and *Entamoeba* genomes encode NMT homologs (AmoebaDB identification numbers [IDs] EHI_159730 and EIN_281920) (24). However, any off-target effect is likely to be limited with the conditions used in the present study, because E. invadens NMT was constitutively transcribed at the trophozoite and early encysting stages and transcription was only downregulated following the encysting stage (Fig. S5). Consistently, trophozoite proliferation was not significantly affected by D-B-09 (IC_50_ of >100 μM). Therefore, D-B-09 is validated to be a useful chemical to elucidate the molecular mechanism underlying *Entamoeba* encystation, in which chitinase participates. D-B-09 also overcomes the limited availability of inhibitors like allosamidin. However, D-B-09 is toxic to human cell lines (25, 26) and may, therefore, not be an appropriate lead compound for developing new drugs against amoebiasis.

E. invadens encysting-cell-associated chitinase activity increased with time during cyst formation, reached a plateau at 72 h postinduction, and was maintained at a high level up to 120 h postinduction. This activity profile is consistent with previous studies using cell homogenates (17). Our results are also consistent with previous mass spectroscopy analyses of purified E. histolytica and E. invadens cyst walls, which showed a relative abundance of chitinase (11, 27). Interestingly, although the transcription of all E. invadens chitinase genes was tightly upregulated at 16 h, the chitinase activity was maintained for 120 h. The different profiles for transcript levels and activities can be explained by chitinase’s stability in the cyst wall, which is supported by recombinant EiCht1 and -4 retaining their specific activities after >3 months of storage at 4°C. Stabilization by association with other cyst wall component(s) is also possible. Taken together, the accumulated evidence indicates that chitinase activity is needed to form the rigid, round cyst wall that confers dormancy to the *Entamoeba* parasite. Chitinase is, therefore, a promising target for the development of new amoebiasis transmission-blocking drugs. However, the molecular mechanism by which *Entamoeba* chitinase contributes to the formation of the complete cyst wall and the possible involvement of chitinase in other encystation processes remain to be addressed.

A necessary characteristic for *Entamoeba* cysts to remain dormant is impenetrability to small molecules (4, 7, 8). A complex of chitin fibrils and a chitin binding protein, Jessie 3, was suggested to be responsible for cyst wall impenetrability (13). The present study shows the importance of chitinase to the rigid structure of the cyst wall; therefore, it is very likely that chitinase is also involved in generating an impenetrable cyst wall. In addition, during cyst formation, the *Entamoeba* plasma membrane becomes enriched with dihydroceramide (Cer-NDS), containing very long *N*-acyl chains (≥26 carbons), which is impermeable to small molecules (9). During encystation, very-long-chain Cer-NDS synthesis and the elevation of chitinase activity both increase sharply between 16 and 24 h and are maintained at high levels from 24 h onward. We therefore assume that plasma membrane impermeability and cyst wall impenetrability to small molecules are cooperatively established by enrichment of Cer-NDS, containing very long *N*-acyl chains (≥26 carbon), and rigid, round chitin wall formation, respectively.

In conclusion, the present study presents distinct lines of evidence to show that chitinase activity is needed to form a mature cyst wall that confers dormancy to the *Entamoeba* parasite, an essential characteristic for this parasite to be transmitted to a new host. In addition, the evidence enables us to propose *Entamoeba* cyst wall formation pathways that involve chitinase (Fig. 5). Furthermore, this study shows chitinase to be a target for the development of new amoebiasis transmission-blocking drugs and that a validated *Entamoeba* chitinase inhibitor would be useful not only for a thorough understanding of chitinase physiology but also for the study of *Entamoeba* encystation.

## MATERIALS AND METHODS

### Materials.

4-Methylumbelliferyl β-d-*N*,*N*′,*N*″-triacetylchitotrioside [4MU-(GlcNAc)^3^], a fluorogenic substrate used for the chitinase assay, was purchased from Sigma-Aldrich (catalog no. M5639; St. Louis, MO, USA), dissolved in 100% DMSO at 1 mM, and stored as stock solution at −30°C. 4-Methylumbelliferone (4-MU), a standard used for activity measurement, was from Tokyo Chemical Industry Co., Ltd. (catalog no. M0453) and stored as a 1 mM stock solution in DMSO at −30°C. The 400 compounds in the Pathogen Box were obtained from MMV and stored as 1 mM stock solutions as described in reference 20. DDD85646 {2,6-dichloro-4-[2-(1-piperazinyl)-4-pyridinyl]-*N*-(1,3,5-trimethyl-1*H*-pyrazol-4-yl)-benzenesulfonamide}, which is D-B-09 in the Pathogen Box, was purchased from Cayman Chemical (Ann Arbor, MI, USA), dissolved in DMSO at 10 mM as a stock solution, and stored at −30°C.

### Production and purification of recombinant E. histolytica chitinase (rEhCht) and recombinant *E*. *invadens* chitinases 1 to 4 (rEiCht1 to -4).

Plasmid constructions, recombinant *Entamoeba* chitinase production in E. coli, and subsequent enzyme purification followed the procedure for producing His-tagged recombinant enzyme described in reference 28, with slight modification. E. histolytica and E. invadens (IP-1) cDNAs were used to construct plasmids. All primers used for plasmid constructions are listed in Table S3.

To obtain crude enzyme solutions, cell pellets were harvested from 100-ml cultures and then resuspended in 4 ml CelLytic B cell lysis reagent (catalog no. B7435; Sigma-Aldrich) supplemented with 80 μl lysozyme (catalog no. L6876; Sigma-Aldrich) (10 mg/ml deionized water [wt/vol]), 8 μl Benzonase nuclease (25 U/μl) (>99% purity; Merck, Kenilworth, NJ, USA), and protease inhibitor cocktail (PIC) (Roche, Basel, Switzerland) in a 5-ml tube and incubated at ambient temperature for 15 min with gentle mixing using a rotator. The cell lysate was then centrifuged at 16,000 × *g* for 10 min at ambient temperature, and the collected supernatant used as crude enzyme solution. All recombinant *Entamoeba* chitinases were eluted in 250 mM imidazole from a prepacked nickel-charged resin column, the His GraviTrap (1 ml bed volume), from GE Healthcare Life Sciences (Buckinghamshire, UK). Purified chitinases were stored at 4°C until use.

### Establishment of a 96-well-plate-based chitinase activity assay.

A chitinase activity assay was performed using a fluorogenic substrate as previously described (23, 29) with slight modification. In detail, a 96-well culture plate, each well of which contained 80 μl 0.15 M citrate-phosphate buffer (pH 7.0) and 10 μl enzyme solution, was preincubated for 5 min either at 37°C for rEhCht or at 26°C for rEiCht1 to -4. Note that the enzyme solution was adjusted to 10 μl by mixing each purified chitinase with 50 mM Tris/HCl buffer (pH 8.0). Upon the addition of 10 μl 100 μM 4MU-(GlcNAc)^3^, which was freshly diluted from the stock solution with deionized water, to each well and mixing, the plate was incubated for the indicated period. The enzyme reactions proceeded at 37°C for rEhCht and at 26°C for rEiCht1 to -4, and the reactions were stopped using 10 μl 3 M sodium carbonate. The plate was then scanned using an EnVision plate reader (PerkinElmer, Inc., Waltham, MA, USA) to measure fluorescence using excitation at 355 nm and emission at 450 nm. To convert the detected signal to the product (4-MU) concentration, a calibration curve of fluorescence signals from 4-MU standard solutions was prepared. For controls, in place of purified chitinase solution (rEhCht or rEiCht1 to -4), 10 μl of a blank (50 mM Tris/HCl buffer [pH 8.0]), a solvent (elution buffer for affinity chromatography), or a mock (empty vector control) solution was added to the assay system. The activity of each sample was calculated after removing the value of the blank control.

For the inhibition assay, a set of 400 compounds (Pathogen Box), which were all adjusted to a concentration of 1 mM as described in reference 20, were thawed from −30°C storage. Then, 10 μl of each stock solution was distributed into separate wells of a 96-well plate and 90 μl deionized water added and mixed. Ten microliters of each diluted solution (100 μM compound solution) was transferred into a new 96-well plate (OptiPlate-96 F; PerkinElmer, Inc.). As a solvent control, 10% DMSO solution (vol/vol in deionized water) was used in place of the compound solution. Subsequently, 80 μl of a mixture of 0.15 M citrate-phosphate buffer (pH 7.0) and purified enzyme solution (0.2 to 1.5 μl) was added to each well. The resulting plates were used in the above-described chitinase activity assay. The final concentrations of each compound and DMSO in a well were 10 μM and 1% (vol/vol), respectively.

To determine IC_50_s, the 10 mM stock solution of D-B-09 prepared as described above was diluted appropriately with DMSO. The volume of each diluted solution was set at 1 μl in a well containing 100 μl reaction mixture; the final DMSO concentration in any well was 1% (vol/vol). As an inhibitor-free control, DMSO solution was used.

### Parasite culture, induction of encystation, and flow cytometry for trophozoite proliferation and cyst formation assays.

Maintenance of E. invadens (IP-1) in routine cultures, induction of encystation, and time course sampling at the indicated time points were performed essentially as described previously (22), except for differences in the conditions and serum concentrations used in encystation-inducing cultures. For testing different parasite conditions, stationary-phase and log-phase trophozoites were prepared for the encystation-inducing culture. The stationary-phase trophozoites were from cultures inoculated with 1 × 10^4^ cells/ml and then incubated for 5 days to a cell density of ∼2.4 × 10^5^ cells/ml. The log-phase trophozoites were from cultures inoculated with 3 × 10^3^ cells/ml and then incubated for 5 days, reaching a cell density of ∼1 × 10^5^ cells/ml. For testing different serum concentrations, encystation-inducing cultures were supplemented with 3 or 10% ABS.

E. histolytica and E. invadens trophozoite proliferation assays were performed essentially as described previously (20) except that in addition to E. histolytica, E. invadens was used and the initial cell number and culture volume were changed. In detail, E. histolytica and E. invadens trophozoites from routine cultures were seeded at 5 × 10^4^ or 6 × 10^5^ cells/240 μl assay medium/well, respectively, in 96-well culture plates.

The E. invadens cyst formation assay was performed essentially as described previously (20) except that stationary-phase and log-phase trophozoites were induced and incubated in 3% or 10% ABS-supplemented encystation-inducing culture for 120 h.

### Native chitinase activity assay.

Cell pellets from 36 wells of a 96-well plate in which encystation was induced were collected at the indicated times in Fig. 2B in a single 5-ml tube using 4 ml phosphate-buffered saline (PBS) and then centrifuged at 770 × *g* for 3 min at 4°C. The cell pellet was washed twice with 4 ml PBS. The cell pellet was then suspended in 518 μl assay buffer for chitinase activity measurement. Note that the final cell density in the assay buffer can be deduced to be 1 × 10^7^ cells/ml because the initial inoculum of the encystation-induced culture was set at 1.44 × 10^5^ cells/well.

### Monitoring the mRNA profile of chitinase during *E*. *invadens* cyst formation.

Cell pellets from two wells of a 24-well plate in which encystation was induced were collected at the indicated times into a single 15-ml tube using 10 ml PBS and then centrifuged at 770 × *g* for 5 min at 4°C. The cell pellet was washed with 6 ml PBS and resuspended in 4 ml PBS. One milliliter of the cell suspension was then dispensed into each of four 1.5-ml tubes, cells were repelleted by centrifugation, and the cell pellets suspended in 1 ml RNAiso plus (TaKaRa). Total RNA extraction, cDNA synthesis, and real-time reverse transcription-quantitative PCR (qRT-PCR) were performed as previously described with appropriate primer sets (Table S3) (9).

### Fluorescence, confocal, and electron microscopy.

E. invadens trophozoites induced for encystation were suspended in encystation medium containing either 10 μM D-B-09 or the solvent control, DMSO. The cell suspensions were then seeded into 96-well culture plates, sealed, and incubated at 26°C for the periods indicated, as described above. Medium containing D-B-09 was prepared by adding 10 mM stock solution at 1/1,000 (vol/vol); therefore, the DMSO content in all wells was 0.1% (vol/vol).

For fluorescence microscopy, cell pellets from two wells of a 96-well plate in which encystation was induced were collected at the indicated times in a single 1.5-ml tube using 0.3 ml PBS and then centrifuged at 770 × *g* for 1 min at ambient temperature. Cell pellets were then resuspended in 300 μl calcofluor white stain (Sigma-Aldrich) diluted 5-fold with PBS or 10 μg/ml FITC-conjugated WGA (Sigma-Aldrich) solution (in PBS). Calcofluor white stain is a premixed solution of calcofluor and Evans blue, and the final concentrations of each were 0.2 and 0.1 mg/ml, respectively. For double staining, 25 μg/ml calcofluor (Sigma-Aldrich) solution (in PBS) was used in place of calcofluor white stain because the fluorescence spectrum of Evans blue partly overlaps that of FITC. The cell suspensions were held for 15 min at ambient temperature and then pelleted by centrifugation at 770 × *g* for 1 min, washed with 500 μl PBS, and then pelleted again. Cell suspensions in PBS were examined under a fluorescence microscope (Zeiss Axio Imager 2; Carl Zeiss, Germany) equipped with a Zeiss AxioCam 305 mono camera (Carl Zeiss). Images were processed using Zen software (Carl Zeiss). In parallel, the samples stained by calcofluor white stain were used for counting the three types of cyst wall structure: pot-like, irregularly shaped, and round. These structures were manually counted until the total counts reached 200 in randomly selected fields under a fluorescence microscope.

For confocal microscopy, samples prepared for double staining were examined by using a confocal laser scanning microscope (Zeiss LSM 880; Carl Zeiss). Image processing and 3-D model construction were done using Zen software (Carl Zeiss).

For transmission electron microscopy, cells pelleted in 96-well plates were treated and processed and data analyzed as previously described (22) except that a JEM-1400Flash was used in place of a JEM-1230 (JEOL, Tokyo, Japan).

## References

[1] Watanabe K, Petri WA, Jr. 2015. Molecular biology research to benefit patients with Entamoeba histolytica infection. Mol Microbiol 98:208–217. doi:10.1111/mmi.13131. 26173474

[2] Ralston KS, Petri WA. 2011. The ways of a killer: how does Entamoeba histolytica elicit host cell death? Essays Biochem 51:193–210. doi:10.1042/bse0510193. 22023450

[3] Carrero JC, Reyes-López M, Serrano-Luna J, Shibayama M, Unzueta J, León-Sicairos N, de la Garza M. 2020. Intestinal amoebiasis: 160 years of its first detection and still remains as a health problem in developing countries. Int J Med Microbiol 310:151358. doi:10.1016/j.ijmm.2019.151358. 31587966

[4] Mi-Ichi F, Yoshida H, Hamano S. 2016. Entamoeba encystation: new targets to prevent the transmission of amebiasis. PLoS Pathog 12:e1005845. doi:10.1371/journal.ppat.1005845. 27764256 PMC5072687

[5] Eichinger D. 1997. Encystation of entamoeba parasites. Bioessays 19:633–639. doi:10.1002/bies.950190714. 9230696

[6] Mitra BN, Pradel G, Frevert U, Eichinger D. 2010. Compounds of the upper gastrointestinal tract induce rapid and efficient excystation of Entamoeba invadens. Int J Parasitol 40:751–760. doi:10.1016/j.ijpara.2009.11.012. 20018192 PMC2881592

[7] Samuelson J, Robbins P. 2011. A simple fibril and lectin model for cyst walls of Entamoeba and perhaps Giardia. Trends Parasitol 27:17–22. doi:10.1016/j.pt.2010.09.002. 20934911 PMC3014499

[8] Moonah SN, Jiang NM, Petri WA, Jr. 2013. Host immune response to intestinal amebiasis. PLoS Pathog 9:e1003489. doi:10.1371/journal.ppat.1003489. 23990778 PMC3749964

[9] Mi-Ichi F, Ikeda K, Tsugawa H, Deloer S, Yoshida H, Arita M. 2021. Stage-specific de novo synthesis of very-long-chain dihydroceramides confers dormancy to Entamoeba parasites. mSphere 6:e00174-21. doi:10.1128/mSphere.00174-21. 33731470 PMC8546694

[10] Samuelson J, Bushkin GG, Chatterjee A, Robbins PW. 2013. Strategies to discover the structural components of cyst and oocyst walls. Eukaryot Cell 12:1578–1587. doi:10.1128/EC.00213-13. 24096907 PMC3889564

[11] Van Dellen KL, Chatterjee A, Ratner DM, Magnelli PE, Cipollo JF, Steffen M, Robbins PW, Samuelson J. 2006. Unique posttranslational modifications of chitin-binding lectins of Entamoeba invadens cyst walls. Eukaryot Cell 5:836–848. doi:10.1128/EC.5.5.836-848.2006. 16682461 PMC1459681

[12] Ghosh SK, Van Dellen KL, Chatterjee A, Dey T, Haque R, Robbins PW, Samuelson J. 2010. The Jacob2 lectin of the Entamoeba histolytica cyst wall binds chitin and is polymorphic. PLoS Negl Trop Dis 4:e750. doi:10.1371/journal.pntd.0000750. 20652032 PMC2907411

[13] Chatterjee A, Ghosh SK, Jang K, Bullitt E, Moore L, Robbins PW, Samuelson J. 2009. Evidence for a “wattle and daub” model of the cyst wall of entamoeba. PLoS Pathog 5:e1000498. doi:10.1371/journal.ppat.1000498. 19578434 PMC2698119

[14] Van Dellen K, Ghosh SK, Robbins PW, Loftus B, Samuelson J. 2002. Entamoeba histolytica lectins contain unique 6-Cys or 8-Cys chitin-binding domains. Infect Immun 70:3259–3263. doi:10.1128/IAI.70.6.3259-3263.2002. 12011021 PMC127964

[15] Frisardi M, Ghosh SK, Field J, Van Dellen K, Rogers R, Robbins P, Samuelson J. 2000. The most abundant glycoprotein of amebic cyst walls (Jacob) is a lectin with five Cys-rich, chitin-binding domains. Infect Immun 68:4217–4224. doi:10.1128/IAI.68.7.4217-4224.2000. 10858239 PMC101730

[16] Villagómez-Castro JC, López-Romero E. 1996. Identification and partial characterization of three chitinase forms in Entamoeba invadens with emphasis on their inhibition by allosamidin. Antonie Van Leeuwenhoek 70:41–48. doi:10.1007/BF00393568. 8836440

[17] Villagómez-Castro JC, Calvo-Méndez C, López-Romero E. 1992. Chitinase activity in encysting Entamoeba invadens and its inhibition by allosamidin. Mol Biochem Parasitol 52:53–62. doi:10.1016/0166-6851(92)90035-i. 1625707

[18] Makioka A, Kumagai M, Hiranuka K, Kobayashi S, Takeuchi T. 2011. Different structure and mRNA expression of Entamoeba invadens chitinases in the encystation and excystation. Parasitol Res 109:417–423. doi:10.1007/s00436-011-2270-2. 21286750

[19] de la Vega H, Specht CA, Semino CE, Robbins PW, Eichinger D, Caplivski D, Ghosh S, Samuelson J. 1997. Cloning and expression of chitinases of Entamoebae. Mol Biochem Parasitol 85:139–147. doi:10.1016/s0166-6851(96)02817-4. 9106188

[20] Mi-Ichi F, Miyake Y, Tam VK, Yoshida H. 2018. A flow cytometry method for dissecting the cell differentiation process of Entamoeba encystation. Front Cell Infect Microbiol 8:250. doi:10.3389/fcimb.2018.00250. 30087858 PMC6066566

[21] Ghosh SK, Field J, Frisardi M, Rosenthal B, Mai Z, Rogers R, Samuelson J. 1999. Chitinase secretion by encysting Entamoeba invadens and transfected Entamoeba histolytica trophozoites: localization of secretory vesicles, endoplasmic reticulum, and Golgi apparatus. Infect Immun 67:3073–3081. doi:10.1128/IAI.67.6.3073-3081.1999. 10338523 PMC96624

[22] Mousa EAA, Sakaguchi M, Nakamura R, Abdella OH, Yoshida H, Hamano S, Mi-Ichi F. 2020. The dynamics of ultrastructural changes during Entamoeba invadens encystation. Parasitology 147:1305–1312. doi:10.1017/S0031182020001079. 32660674 PMC10317766

[23] Dey T, Basu R, Ghosh SK. 2009. Entamoeba invadens: cloning and molecular characterization of chitinases. Exp Parasitol 123:244–249. doi:10.1016/j.exppara.2009.07.008. 19646441

[24] Frearson JA, Brand S, McElroy SP, Cleghorn LA, Smid O, Stojanovski L, Price HP, Guther ML, Torrie LS, Robinson DA, Hallyburton I, Mpamhanga CP, Brannigan JA, Wilkinson AJ, Hodgkinson M, Hui R, Qiu W, Raimi OG, van Aalten DM, Brenk R, Gilbert IH, Read KD, Fairlamb AH, Ferguson MA, Smith DF, Wyatt PG. 2010. N-myristoyltransferase inhibitors as new leads to treat sleeping sickness. Nature 464:728–732. doi:10.1038/nature08893. 20360736 PMC2917743

[25] Müller J, Aguado A, Laleu B, Balmer V, Ritler D, Hemphill A. 2017. In vitro screening of the open source Pathogen Box identifies novel compounds with profound activities against Neospora caninum. Int J Parasitol 47:801–809. doi:10.1016/j.ijpara.2017.06.002. 28751177

[26] Kallemeijn WW, Lueg GA, Faronato M, Hadavizadeh K, Goya Grocin A, Song OR, Howell M, Calado DP, Tate EW. 2019. Validation and invalidation of chemical probes for the human N-myristoyltransferases. Cell Chem Biol 26:892–900.e4. doi:10.1016/j.chembiol.2019.03.006. 31006618 PMC6593224

[27] Ali IK, Haque R, Siddique A, Kabir M, Sherman NE, Gray SA, Cangelosi GA, Petri WA, Jr. 2012. Proteomic analysis of the cyst stage of Entamoeba histolytica. PLoS Negl Trop Dis 6:e1643. doi:10.1371/journal.pntd.0001643. 22590659 PMC3348168

[28] Mi-Ichi F, Ishikawa T, Tam VK, Deloer S, Hamano S, Hamada T, Yoshida H. 2019. Characterization of Entamoeba histolytica adenosine 5′-phosphosulfate (APS) kinase; validation as a target and provision of leads for the development of new drugs against amoebiasis. PLoS Negl Trop Dis 13:e0007633. doi:10.1371/journal.pntd.0007633. 31425516 PMC6715247

[29] Muñoz PLA, Minchaca AZ, Mares RE, Ramos MA. 2016. Activity, stability and folding analysis of the chitinase from Entamoeba histolytica. Parasitol Int 65:70–77. doi:10.1016/j.parint.2015.10.006. 26526675

