# Supplementary material for: Entamoeba Chitinase is Required for Mature Round Cyst Formation
Source: Microbiol Spectr. 2021 Aug 4;9(1):10.1128/spectrum.00511-21. doi: 10.1128/spectrum.00511-21 (PMC8552601; doi:10.1128/spectrum.00511-21)
Supplement: SUPPLEMENTAL FILE 2 — Supplemental material. Download SPECTRUM00511-21_Supp_2_seq3.pdf, PDF file, 1.5 MB [file spectrum00511-21_supp_2_seq3.pdf]

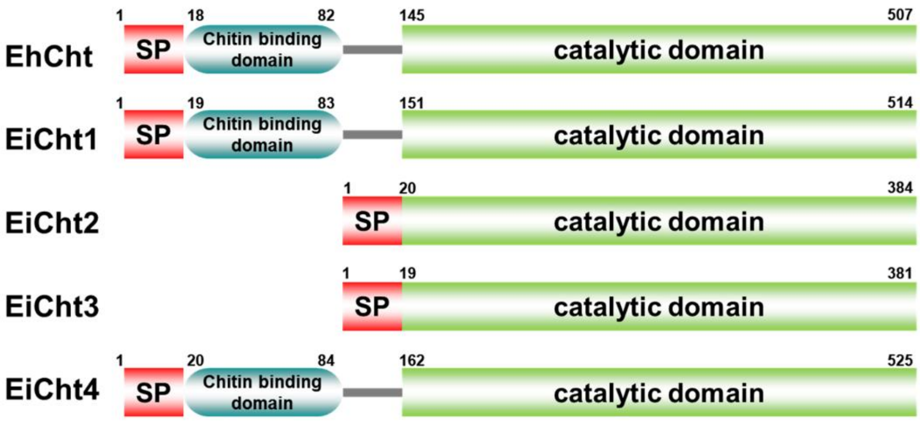

Figure S1. Schematic structures of *Entamoeba* chitinases. All *Entamoeba* chitinases have a conserved catalytic domain. EhCht, and EiCht1 and 4 also have a conserved 60-amino acid N-terminal 8-Cys CBD that is unique to *Entamoeba* (1) . Numbers indicate the amino acid sequence number. SP, signal peptide.

rEhCht-plate A

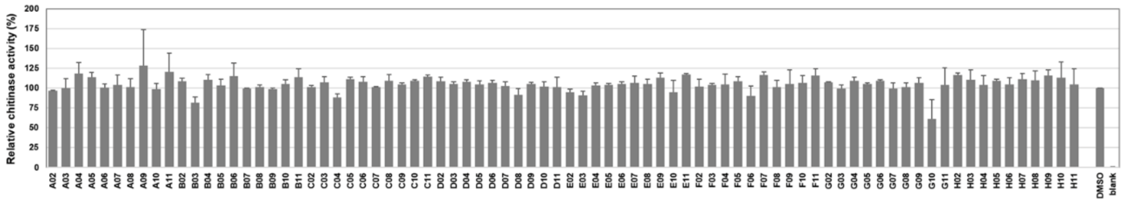

rEiCht1-plate A

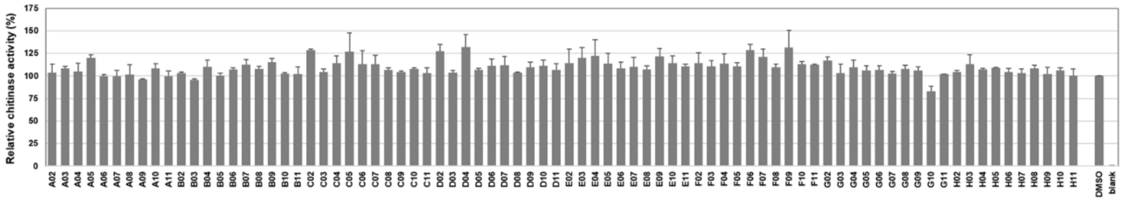

rEiCht4-plate A

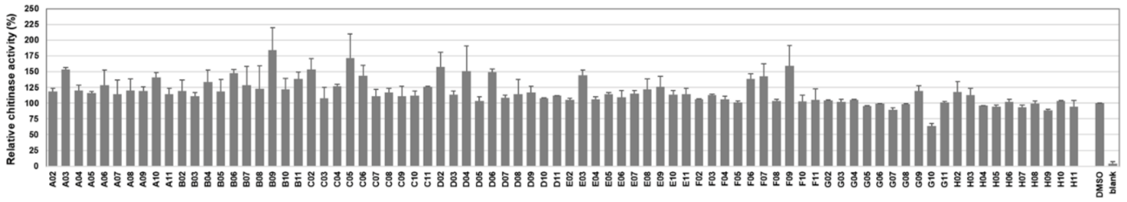

rEhCht-plate B

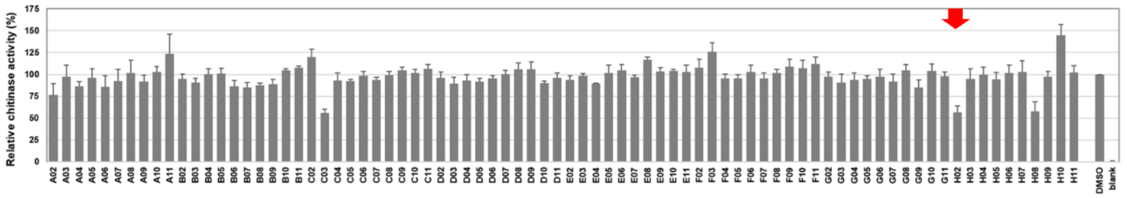

rEiCht1-plate B

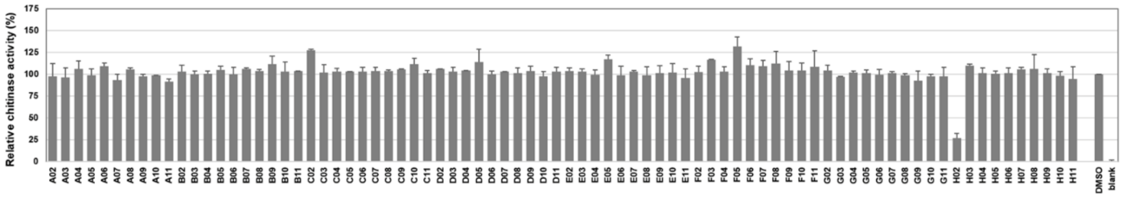

rEiCht4-plate B

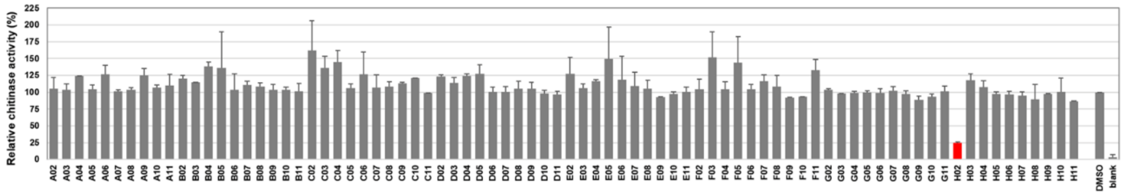

rEhCht-plate C

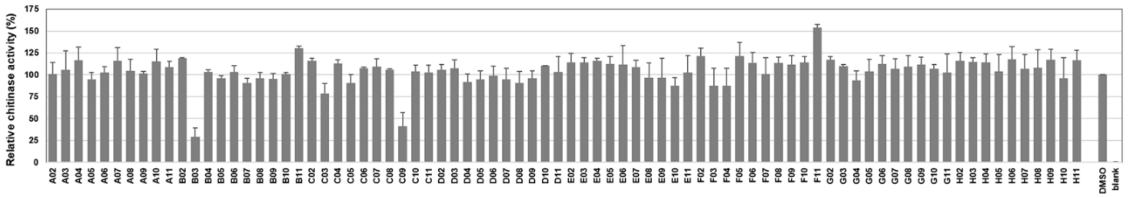

rEiCht1-plate C

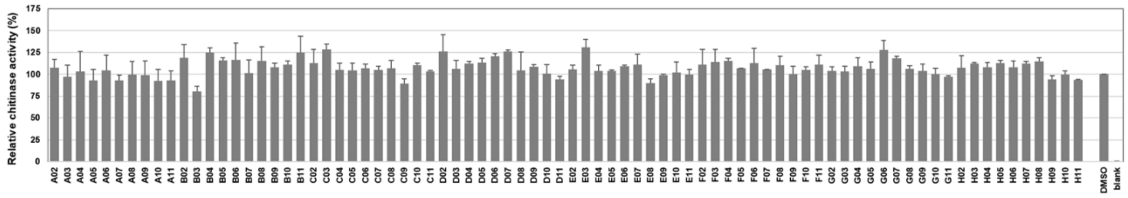

rEiCht4-plate C

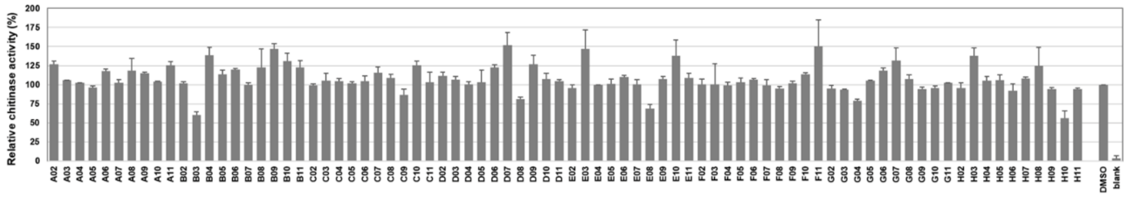

rEhCht-plate D

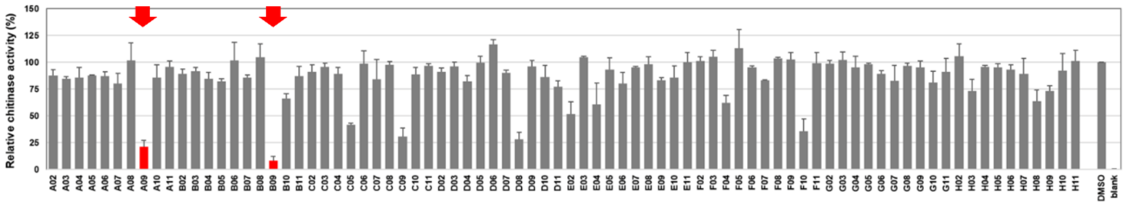

rEiCht1-plate D

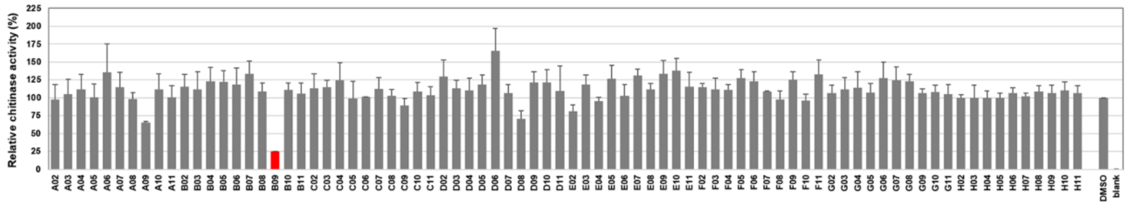

rEiCht4-plate D

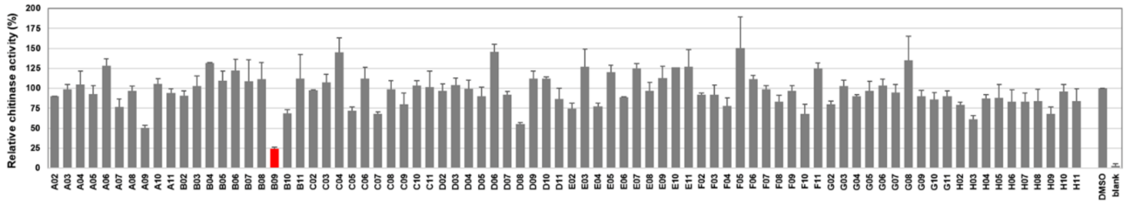

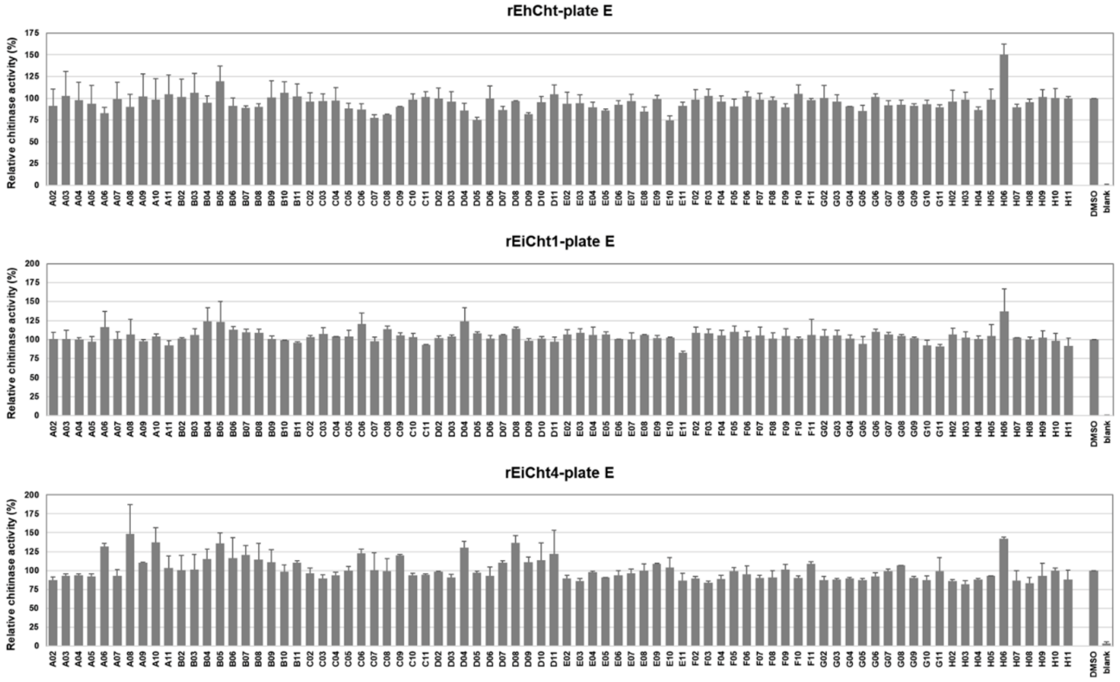

Figure S2. Screening 400 compounds with diverse scaffolds from the MMV Pathogen Box by the *in vitro* chitinase activity assay. (A–E) The effect(s) on activities of rEhCht (upper), rEiCht1 (middle) and r EiCht4 (lower). Data are expressed as the residual activity expressed as the percentage of the activity in each sample relative to that in the DMSO control (set as 100%). Data are shown as the mean with error bar (deviation) from two independent experiments. Red arrows indicate compounds that reproducibly inhibited at least one chitinase activity by ≥75%. DMSO and blank controls were included. Five 96-well plates (plates A–E) were used, into which 400 compounds together with DMSO and blank controls were equally dispensed (80 wells per plate).

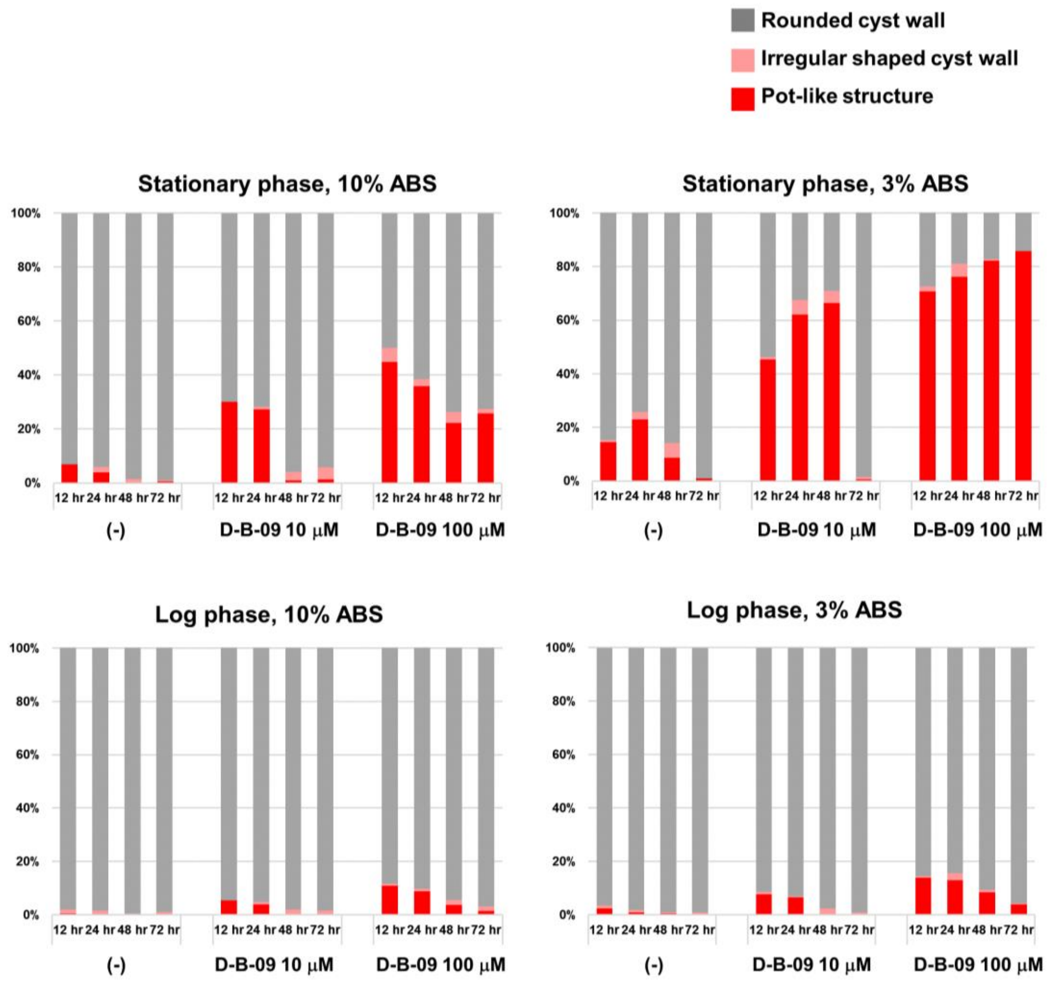

Figure S3. Stacked bar graph of percentages of round, irregular shaped and pot-like cyst walls. Effects of encystation induction conditions and serum concentrations in the encystation-inducing culture were analyzed at 12, 24, 48, and 72 h post induction. Representative data are shown from two independent experiments.

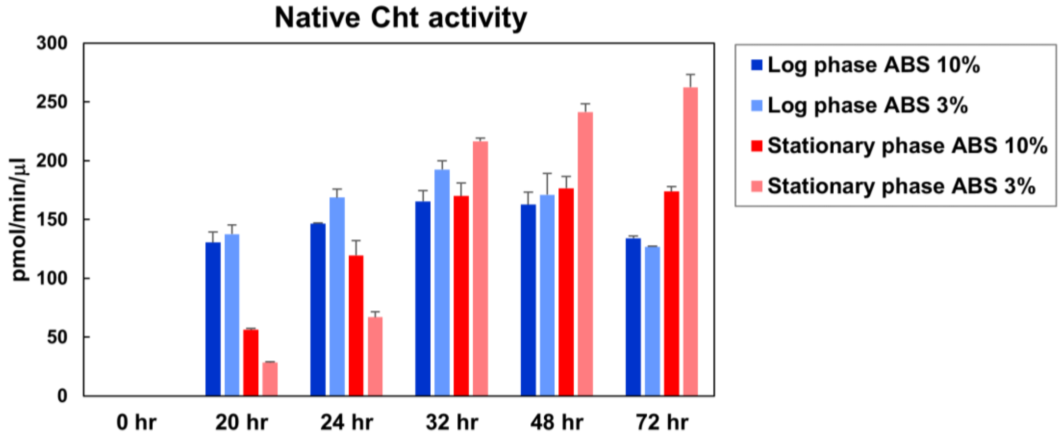

Figure S4. Time course of *E. invadens* cell-associated chitinase activity in encystation-inducing cultures using different induction conditions and serum concentrations. Representative data are shown from two independent experiments.

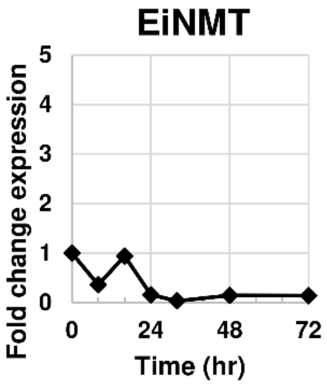

Figure S5. Changes in *EiNMT* gene transcription during encystation. Expression levels are shown as fold changes at the indicated time points after the induction of encystation relative to the level at time 0 h. Representative data are shown from three independent experiments.

Table S1. Encystation conditions affect efficiency of cyst formation and sensitivity to D-B-09. IC_{50} values were determined from three independent experiments and are expressed as the average ± SD.

	Encystation rate			
**Parasite condition**	**Stationary phase**		**Log phase**	
**ABS concentration**	**10%**	**3%**	**10%**	**3%**
**D-B-09, IC_{50} (120 hr)**	**~100 μM**	**1.4 ± 0.3 μM**	**>100 μM**	**>100 μM**

Table S2. Encystation conditions do not affect native chitinase activity in response to D-B-09. IC_{50} values were determined from two independent experiments and are expressed as the average ± deviation.

	Native chitinase activity			
**Parasite condition**	**Stationary phase**		**Log phase**	
**ABS concentration**	**10%**	**3%**	**10%**	**3%**
**D-B-09, IC_{50}**	**0.4 ± 0.1 μM**	**0.5 ± 0.1 μM**	**0.5 ± 0.1 μM**	**0.6 ± 0.1 μM**

Table S3. List of primers.

Described section of Method	Target gene	Name	Sequence	Direction	Mention Underline
Production of recombinant *Entamoeba* chitinase	EhCht	Pc-EhCht-F	5'-ATCGGTACCCACAACTGTGAAGGTCTTTC-3'	sense	KpnI restriction site
		Pc-EhCht-R	5'-ATCCTGCAGTTAACATTTCTCAATTAGACTCTTAA-3'	antisense	PstI restriction site
	EiCht1	Pc-EiCht1-F	5'-ATCGGTACCTCACAAACATGTGAAGGACTC-3'	sense	KpnI restriction site
		Pc-EiCht1-R	5'-ATCCTGCAGTTAGCACTGATCAAGCTCTTTTTTG-3'	antisense	PstI restriction site
	EiCht2	Pc-EiCht2-F	5'-ATCGGTACCATGGGATTGGAAGTTTTAAGAAAG-3'	sense	KpnI restriction site
		Pc-EiCht2-R	5'-ATCCTGCAGTCAATCAGATGCTAAAACACTTCTG-3'	antisense	PstI restriction site
	EiCht3	Pc-EiCht3-F	5'-ATCGGTACCCAAAAAGAGTATAAAGTGATTGG-3'	sense	KpnI restriction site
		Pc-EiCht3-R	5'-ATCCTGCAGTTAGTTTTTCAACTCATCGATC-3'	antisense	PstI restriction site
	EiCht4	Pc-EiCht4-F	5'-ATCGGTACCCATTCACAAACATGCGAAG-3'	sense	KpnI restriction site
		Pc-EiCht4-R	5'-ATCCTGCAGTTAACATTTGTCGATTTGTTTTTTG-3'	antisense	PstI restriction site
qRT-PCR	EiCht1	QT-EiCht1-F	5'-CTGGGTTGGTGTGCAAGTGT-3'	sense	
		QT-EiCht1-R	5'-CCTCCGACGAAGACTCTGCT-3'	antisense	
	EiCht2	QT-EiCht2-F	5'-GCAAACGCCAAAACAAAACA-3'	sense	
		QT-EiCht2-R	5'-GCCTGGGTATTCCCAATCAA-3'	antisense	
	EiCht3	QT-EiCht3-F	5'-GACGTCCTCGTCGAAGGAAT-3'	sense	
		QT-EiCht3-R	5'-CATCGTCCGAGTCGTGGTAA-3'	antisense	
	EiCht4	QT-EiCht4-F	5'-GCTTACAATCGCAGCACCAG-3'	sense	
		QT-EiCht4-R	5'-CAGGCGCCATGTAAATCGTA-3'	antisense	
	EiNMT	QT-EiNMT-F	5'-GCGCAATACTTCCATCGCCC-3'	sense	
		QT-EiNMT-R	5'-GCACGCTGCATCGTCAATCT-3'	antisense	
	EiRNApol	QT-EiRNApol-F	5'-GTTCTGTCCGCAATGTCAGC-3'	sense	
		QT-EiRNApol-R	5'-TTGGGGTCCTTCCATTTTTC-3'	antisense	

Movie S1. Three dimensional models for pot-like structures stained with calcofluor (blue) and FITC-conjugated WGA (green).

Reference

1. Samuelson J, Robbins P. 2011. A simple fibril and lectin model for cyst walls of Entamoeba and perhaps Giardia. Trends Parasitol 27:17-22.
